# “I have to charge my social battery”: Perspectives from autistic young adults on Quality of Life

**DOI:** 10.1177/13623613241245578

**Published:** 2024-04-17

**Authors:** Elisabeth Øverland, Åshild Lappegard Hauge, Stian Orm, Merete Glenne Øie, Erik Winther Skogli, Elizabeth Pellicano, Per Normann Andersen

**Affiliations:** 1 Innlandet Hospital Trust, Norway; 2 Inland Norway University of Applied Sciences, Norway; 3 University of Oslo, Norway; 4 University College London, UK; 5 Macquarie University, Australia

**Keywords:** autism, emerging adulthood, quality of life, reflexive thematic analysis

## Abstract

Several studies report on lower quality of life for autistic people when compared to non-autistic people. However, there are ongoing discussions about the conceptualization of quality of life for the autistic population. This qualitative study investigated self-perceived quality of life in the context of autistic young adults’ everyday lives. Participants (age range 21–29 years) were recruited from a 10-year follow-up study, Lillehammer Neurodevelopmental Follow-up Study, all diagnosed in childhood and adolescence. Fourteen individual in-depth interviews were conducted. When asked about what was important for having a good quality of life, our autistic participants described relationships to people and pets as important, as well as having meaningful activities. Specific interests had guided many to their choice of both academic and work career. Many also described obstacles in their environment that had influenced their quality of life negatively, such as being misunderstood by teachers and other professionals, being bullied, and sensory and emotional overload. Our findings indicate that those in supportive roles should provide help with establishing connections to activities and people/animals, as this seems to be essential for creating a good quality of life. Taking advantage of passions and interests can create opportunities for autistic people. Findings suggest that future research should address the communication barriers between autistic people and professionals that can lead to misunderstandings.

**Lay abstract:**

In this study we have asked a group of autistic young adults to describe what is important for their quality of life. The 14 participants (aged 21–29 years) were recruited from a 10-year follow-up study of autistic people. During interviews, our participants described the importance of having relationships with family, friends and pets. Having meaningful activities and being able to immerse themselves in particular interests was also reported to be important for a good quality of life. Interests had also guided their choice of what to study and what to do for work. They also spoke of how communication problems with professionals, bullying and sensory and emotional overload could have a negative impact on quality of life. Future interventions should focus on how professionals can help autistic people to connect to people/animals and meaningful activities, as the participants described this as important for having a good quality of life. These findings may be helpful in enhancing how passions and interests can be seen as opportunities for both academic and work careers for autistic people. Future research and interventions should also look at the communication barriers between autistic people and professionals, and how two-way understanding can be improved.

## Introduction

Historically, the majority of autism research has been informed by the medical and psychiatric field, where autism is often described as a collection of “deficits” needing to be “fixed” (Hogan, 2019, p. 9). During the last two decades, both researchers and the autistic community itself have raised concerns about this overly deficits-focused approach to understanding autism (Milton & Sims, 2016; Pukki et al., 2022; Robertson, 2010). There is now a gradual shift from seeking to understand autism by observable signs and “symptoms” alone to first-person, subjective experiences. As a result, there has been an increasing interest in exploring quality of life (QoL) for the autistic population (Evers et al., 2022). More knowledge about perceived QoL in the autistic population could help influence how support and services can facilitate improvement in everyday life (McConachie et al., 2020).

QoL refers to an individual’s subjective perception of the quality of their health, relationships, school/job satisfaction within the context of the culture, and value systems in which they live (World Health Organization (WHO), 1998). QoL can be conceptualized through both *objective* dimensions such as facts about income, age, sex, living conditions, health, education, and *subjective* dimensions, such as evaluation about life satisfaction or well-being (Barstad et al., 2016; Cummins, 2000; Eurostat, 2015). Relying on the findings from multiple researchers over 30 years, Schalock (2000, 2004) presented eight core QoL domains referring to the factors that compose personal well-being, including interpersonal relations, social inclusion, personal development, physical well-being, self-determination, material well-being, emotional well-being, and rights. Research has suggested that despite the challenges people in marginalized positions face, outcomes of QoL may be more positively perceived with the use of measurement of subjective person-referenced outcomes, as with these eight core domains (Schalock, 2000; Scott & Sedgewick, 2021; Thompson et al., 2018).

Autism can be described as a different way of sensing and interpreting the world, with specific cognitive strengths and challenges in the domains of language, communication, social interactions, sensory processing, motor skills, and self-regulation (Robertson, 2010). This different way of experiencing the world might indicate that QoL for autistic people might be perceived differently than for non-autistic people. When measured with instruments designed for the non-autistic population, autistic children, adolescents, and adults report poorer QoL throughout their lives when compared to non-autistic adults (Ayres et al., 2018; Baxter et al., 2015; Ikeda et al., 2014). Several studies have found that autistic people face specific challenges that may affect QoL negatively (e.g. de Vries & Geurts, 2015; Graham Holmes et al., 2020). These challenges are connected to high levels of stress, sleep problems, sensory processing differences, mental health problems, executive function difficulties, and social interaction problems (Andersen et al., 2023; Backman et al., 2023; Knüppel et al., 2018; Oakley et al., 2021). Autistic young people are also at much higher risk of experiencing social exclusion and bullying at school (Humphrey & Hebron, 2015; Maiano et al., 2016), which are predictors of poorer perceived QoL (Hong et al., 2016).

Qualitative studies have confirmed the challenges outlined above and suggested an increased risk for poor QoL in the autistic population, especially during transition periods (e.g. Cribb et al., 2019; Sosnowy et al., 2018). One such transition is the period between adolescence and young adulthood (18–25 years), so-called *emerging* adulthood (Arnett, 2000). Emerging adulthood is characterized by identity exploration, instability, self-focus, and a feeling of being in between adolescence and adulthood. Emerging adulthood is also associated with greater levels of depression and suicidality (Schwartz & Petrova, 2019). Transitions often involve uncertainty about what is ahead, and uncertainty can be experienced as more challenging for autistic people than non-autistic people (Leung & Zakzanis, 2014). Poor transition outcomes for autistic young adults, such as limited participation in further education, low rates of independent living, and high unemployment rates, are connected to several intersecting factors (Thompson et al., 2018). These include poor person-environment fit, uncertainty about the role of parents during transitions, and a lack of comprehensive or poorly administered services (Anderson et al., 2018).

Furthermore, the adaptive skills of autistic young adults are often lower than their intellectual capacity (Matthews et al., 2021). This could lead to interdependent living; balancing how to manage daily life obstacles on one’s own, receiving support from formal services, and parents’ involvement (Matthews et al., 2021). The transition from adolescence to adulthood is thus a sensitive period where QoL could be affected negatively in many ways, especially for potentially vulnerable populations. Perceived QoL in this particular period of life is therefore important to investigate.

Various instruments have been used in measuring QoL in autistic populations, and the conceptualization of QoL varies, often mixing subjective and objective factors in outcome measures (Evers et al., 2022). Generic measures often focus on achieving objective, standard life outcomes, which are not necessarily described as meaningful by autistic people themselves (Bishop-Fitzpatrick et al., 2016; Pellicano, Fatima, et al., 2022). Both Schalock (2000) and Evers et al. (2022) argue that by focusing only on subjective dimensions, one could improve the conceptualization of QoL for marginalized groups, including the autistic population. Indeed, there could be autism-specific themes connected to QoL that is not well captured in generic measures, for example, the accessibility to health services (Mason et al., 2018). In trying to capture autism-specific themes, McConachie et al. (2018) have developed a self-report measure called ASQoL. The questions in ASQoL are developed from focus group interviews with autistic people, and covers, for example, barriers connected to accessing health services, sensory issues, and autistic identity.

Concerns have been raised about the use of generic QoL self-report instruments for the autistic population, considering the particular wording and formatting of questions, which may lead to invalid answers. Following these concerns, the validity of self-report measures has been questioned, which has led to recommending the use of proxy reports from caregivers or teachers (Mazefsky et al., 2011). These practices have contributed in making the voices of autistic people less important, which have recently been discussed critically (Pellicano & Heyworth, 2023). It is essential to understand how autistic people perceive a good life themselves, rather than what is constructed by others (Lam et al., 2021).

QoL is a relatively new field in autism research. The period of emerging adulthood is characterized by many transitions and is proven to be a challenging period of life that can affect QoL in many ways. There is a need to explore further how QoL is subjectively perceived and conceptualized among autistic people in this particular period. In this study, we interviewed a clinical sample of autistic young adults without intellectual disabilities that has been followed over 10 years, from childhood to young (emerging) adulthood. We aimed to explore how they described and conceptualized their QoL. Specifically, we asked,

1. What do autistic young adults describe as important for QoL throughout the different phases of childhood, adolescence and especially in the transition to adulthood?
2. What do autistic young adults perceive to be the challenges and barriers to the possibility of having a good QoL?

## Method

### Participants

The participants were recruited from the Lillehammer Neurodevelopmental Follow-up Study (LINEUP) in Norway. In this study, children referred to Innlandet Hospital Trust for assessment of neurodevelopmental disorders were followed from baseline (T1, *M*_age_ = 12 years) through 2-year follow-up (T2, *M*_age_ = 14 years) and 10-year follow-up (T3, *M*_age_ = 22 years). Thirty-eight participants fulfilled *Diagnostic and Statistical Manual of Mental Disorders* (4th ed.; *DSM-IV*) criteria for a diagnosis on the autism spectrum (Asperger syndrome: *n* = 31; PDD-NOS: *n* = 7) at T1. Guided by the concept of “information power” (Malterud et al., 2016), we purposively sampled 19 participants for this interview study (Etikan et al., 2016), as this represented 50% of the group with an autism spectrum diagnosis from T1. Given that there was a skewed gender representation in the sample at T1, we contacted all the females who had consented to further participation in the study (*n* = 4). Of the 19 contacted, 14 (74%; *M*_age_ = 23 years; age range = 21–29) responded positively and were included in the current study. All were in the average range of cognitive ability, measured with Wechsler Abbreviated Scale of Intelligence at T1. Almost all reported being of European-white ethnic background. One participant reported being of mixed race. All but one had completed high school. Most participants lived independently (see Table 1).

**Table 1.** Demographic characteristics.

Gender	*N*	%
Man	10	71
Woman	3	21
Non-binary	1	7
**Daily occupation**		
Student (bachelor or master)	6	42
Full-time job	4	28
On disability benefits	2	14
Work training	1	7
No organized activity	1	7
**Living arrangements**		
Student housing	5	35
Self-cared housing alone	2	14
Self-cared housing with partner	3	21
In parents’ home	3	21
Staffed care home	1	7
Urban living	10	71
Rural living	4	28

### Data collection

All 14 semi-structured interviews were conducted in person by EØ, who is experienced in interviewing people on the autism spectrum. The participants were invited to a place they were comfortable or preferred: a neutral office at the hospital (*n* = 9), a conference or hotel room (*n* = 4), or at their home (*n* = 1). The interviews were conducted in Norwegian, during July and August 2022. Interviews ranged from 56 to 143 min, with an average of 85 min.

We drew on previous existing research to inform the basis of the interview guide (Crompton et al., 2020; Kapp et al., 2019; Lilley et al., 2022). Together with the research group’s autistic member, we invited representatives from the Norwegian Autism Society to participate in collaborative meetings to develop the interview questions, and to take part in an individual pilot interview.

The interview guide was designed to gather participants’ experiences much broader than QoL (see protocol article, Øverland et al., 2022 for full description). The interview topics that mapped on to the research questions considering QoL, included (1) Daily occupation and living conditions; (2) Growing up with autism and how this has influenced QoL throughout the different phases of childhood, adolescence and emerging adulthood, and (3) What is perceived as important for having a good life at present. We included specific probe questions following each main question, although probe questions were only asked if further elaboration was needed (see Supplementary Materials for the full interview schedule). The main questions from the interview guide were sent to the participants 1 week before the interview. The interviews themselves were recorded with participants’ prior consent, and subsequently transcribed verbatim and anonymized.

### Data analysis

We adopted reflexive thematic analysis for this study (Braun & Clarke, 2021). Using a relativist ontology, we see ourselves as researchers who construct the findings through interpretation. In this process, we are cognizant of our pre-existing assumptions, how these influence the interviews with the participants, and the interpretation of the findings (Braun & Clarke, 2021; Malterud, 2001). Our research group consists of people with and without autism. Representatives from the Norwegian Autism Society have participated in this study at different time points. We represent an interdisciplinary group with theoretical and clinical knowledge of autism from training and expertise in psychology, education, and social welfare. Our backgrounds have implications for our theoretical assumptions concerning autism and what it means to be autistic. With a foot in contextualism, we align with the neurodiversity paradigm where “living in a society designed for non-autistic people contributes to, and exacerbates, many of the daily living challenges that autistic people experience” (Robertson, 2010, p. 3). For further discussion of the neurodiversity paradigm, see Pellicano and den Houting (2022).

We followed the six phases of reflexive thematic analysis (Braun & Clarke, 2021), taking an inductive (bottom-up) approach. To familiarize ourselves with the data (Phase 1), all Norwegian authors read the transcripts, taking notes of any recurring elements across the dataset. When reading, we had the research questions in mind but were also open for other elements appearing across the dataset. The first author re-read the transcripts several times and then coded all the transcripts (Phase 2), with an emphasis on semantic (descriptive) content, organized using NVivo 12 for Windows. We further discussed as a team how the codes could fit into a thematic structure and then generated the initial themes (Phase 3). At this point, we added more focus on latent (interpretative) codes and themes. As a result, we developed some new themes and merged others (Phase 4). In line with our epistemological stance, we worked on finding concise and informative names for the themes (Phase 5). After having written up some of the themes by including several quotes, the naming of themes was finally set. The last phase of reflexive thematic analysis, writing up (Phase 6), was been done while writing this article. The quotes used in this article are translated from Norwegian.

### Community involvement

As previously mentioned, representatives from the Norwegian Autism Society were involved in developing the research questions, provided feedback on the interview guide, and participated in a collaborative meeting during the analytic process. Their reflections are included in this article. For example, they guided us to be more inclusive in our language when asking and reporting about what could be described as a family constellation. One of our co-researchers/authors with autism also advised the research group on the wording and approach to the research questions and interview guide, and the interpretation of results.

We use person-first (person with autism) and identity-first (autistic person) language interchangeably in this article. Most of the participants in this study used person-first language when being interviewed, and this is also the preference of the Norwegian Autism Society. We are aware of the preference for identity-first language, especially in English-speaking societies (Bury et al., 2020; Kenny et al., 2016; Taboas et al., 2023). However, there might be language and cultural differences regarding this issue, and we therefore use both terms, as Buijsman et al. (2022) advocate.

## Results

Through our analysis, we identified five themes that addressed our research questions. We show a summary of the themes and subthemes with some illustrative quotes in Table 2. In the following text, the themes are numbered and presented in bold; subthemes in italics.

**Table 2.** Overview of themes and subthemes, with illustrative quotes.

Theme	Subtheme	Illustrative quotes
1. Feeling connected to family and peers	1.1. Family provides safety and security 1.2. Sense of belonging with peer group	“They (parents) were very good at taking it easy with me, really. So, at the same time as I feel safe around them, they teach me everything, really.” “What I am most grateful for, is probably the friends I have close to me (. . .) They push me, give me experiences in life.”
2. Interactions with teachers and practitioners	2.1. Being understood 2.2. Receiving social and academic support at school	“People must ask the right questions. Because my psychologist said, ‘tell me what your problem is’. I didn’t manage to answer that”. “I had someone to help me get an overview of things. Because I get easily frustrated if I lose overview (..) when we changed rooms and things like that. It was quite helpful.”
3. Becoming more in control	3.1. Balancing social energy 3.2. Preferring sensory-friendly environments 3.3. Establishing everyday routines	“It drained a lot on the social battery (. . .) to be with others (. . .) I need to be alone (. . .) then it gradually gets better.” “I noticed at that party that I got tired very quickly and it was too stressful there.” “Routines when it comes to food at day, routines when it comes to work at day, routines when it comes to everything, really.”
4. Deriving meaning and purpose from interests and passions	4.1. Interests as a way of spurring friendships 4.2. Interests as a guide to education and job career 4.3. Finding meaningful activities	“So, I found two friends there, mostly because we were all interested in history.” “I am very interested in politics, very interested in community management (. . .) my dream is to have that as a job.” “I found that it was most important for me to follow what I had an interest in.”
5. Cautious optimism about their futures	5.1. Expanding opportunities in work and social life 5.2. Uncertainty about wanting a family	“I wish for a social life, and if I want that, I have to try having someone close.” “I haven’t thought a lot about [own] family yet. Not in five years, but perhaps in five more years, I don’t know.”

### Theme 1: feeling connected to family and peers

All participants described being connected to either family, animals, and/or peers as important for QoL throughout childhood, adolescence, and emerging adulthood. In various ways, they highlighted that *family provides safety and security (subtheme 1.1).* For most participants, family was described as where they got support, but also where they got pushed and encouraged to participate in social settings, especially through childhood and adolescence. A few described the vulnerability in not having a steady home or having sparse contact with parents. Others expressed frustration of parents being too concerned for their ability to live independently: “my mother . . . has an attitude of me not managing, I feel. And that irritates me.” Interdependence, as described by Matthews et al. (2021) and also found in the study of Elias and White (2018) was also evident with some of our participants. For example, one participant who lived in a student home described intermittent periods when they needed support from family: “I get big problems with just taking care of myself.” When this happened, the participant returned home to their parents, who provided regular routines, food and support so they could recuperate and then return to their student housing. Other participants had tried to live far away from the family home while studying but had soon moved closer to family, as this provided a feeling of security.

Many participants also described their strong connection to pets, which were considered part of the family. We did not ask specifically about pets, but many participants still described their relationship with pets in detail. They described their connection to pets as close and fulfilling, and that communication was more straightforward with animals:

It gives me another peace, another confidence. When I had a horse, I noticed they have very direct body language. If you do something they don’t like, they tell you . . . Humans, we can lie, we can come up with a white lie, we can try to get our way in different ways. We can hide things, exaggerate, make fun. And animals don’t do that.

In addition to family, most participants also reported a desire to be connected and to have a *sense of belonging with their peer group (subtheme 1.2)*. The participants generally described a wish to socialize with others. When asked what was important for living a good life, one participant replied: “For me, I think it is friends, having someone nearby that I can trust and talk to and guide me a bit, push me a little.” Another participant described being asocial as “the worst stigma around autism.” At the same time, being with many people could for some be exhausting, so the balance between being with someone and being alone was important, as described by one participant: “I have a very strong need to be alone . . . but I also have a very strong need to not be too much alone.” A description of a “social battery being emptied” when being overly exposed in social settings was evident for many of the interviewees: “I think my battery just got empty, really, of being social, because there is *so* much going on all the time.”

When describing how they struck this balance when socializing with peers, the participants also reflected on how they perceived themselves as different: “I can get insecure about myself because I am not like all the others.” Feeling different also included not being connected to classmates, not having the same interests as peers, or experiencing mutual misunderstandings in contact with people. Some of the participants connected this to their autism diagnosis: “I was different. Because I was on the spectrum, as we found out later.” Most participants described being bullied in childhood, both verbally and physically. Bullying was also described in more subtle ways: “well, nobody approached me, and when I approached them, I was usually put aside or ignored.” None of the participants described bullying after the transition to senior high school. Also, most participants said they established closer connections to peers and socialized with larger peer groups at senior high school and college/university, usually through shared interests.

### Theme 2: interactions with teachers and practitioners

The participants shared stories about being met by teachers and other healthcare practitioners, often in unfortunate ways. *Being understood (subtheme 2.1)* by people in these helping roles was imperative for QoL. The participants described many instances in which teachers displayed little understanding—or empathy—with their autistic students. For example, one participant remembered asking the teachers repeatedly why the class should do different assignments, and often got the answer “because I tell you to.” The participant was unhappy with such an answer, having a strong need for explanation and prediction. The participant was then considered oppositional by their teachers.

Some described that meeting practitioners who had a reflective and philosophical approach, could lead to frustration: “I felt that they repeated what I said . . . but gave the impression that it was their conclusion, or their input. That really just felt condescending and unproductive.” Hence, the participants expressed a desire for more direct questions and advice for what to do when struggling.

The participants recalled *receiving social and academic support at school (subtheme 2.2)*. Being taught in small groups was common, which was described positively. A few participants described academic difficulties, but social difficulties were more common. Those having social challenges received little support, but in hindsight, wished that school had been more aware of these challenges. Poor social and academic support at school was described by some as leading to less school attendance and mental health problems not being addressed: “Everything that happened, or more correctly, did *not* happen when I was a child and adolescent—it’s pretty hard.”

The participants considered tailored support to be vital for thriving at school. This was evident for quite a few in the transition from junior high (ages 13-16) to senior high school (ages 16-19), as a majority described better opportunities to get academic support at senior high school: “One teacher took me through what I knew and not knew, found gaps in my knowledge and taught me those things . . . Made it much easier.”

### Theme 3: becoming more in control

As described in both Themes 1 and 2, the participants described that they often met environments that did not always understand and accommodate for them. Thus, they were making efforts to protect themselves from being overwhelmed. Many of the participants described having been through a trial-and-error period where they gradually developed various helpful strategies that they now use in their everyday lives. This helped them to become more in control over their own everyday experiences. Quite a few described *balancing social energy (subtheme 3.1)* as essential for coping with daily life. As described in Theme 1, some the participants referred to their “social battery being emptied” and that they needed time by themselves to “recharge.” To balance this, they practiced saying they were busy or postponing appointments to have more time on their own. Participants also frequently described *preferring sensory-friendly environments (subtheme 3.2)*. For example, leaving a noisy party, not using the public reading rooms at university, working with headphones on, or choosing a job that offered permanent working-from-home instead of a job with on-site facilities. These strategies contributed to the “social battery” being uploaded for longer, avoiding awkward social situations and/or disruptive physical environments.

Also, *establishing everyday routines and activities (subtheme 3.3)* helped. Many described difficulties concerning executive function, such as organization of the day and finding motivation for tasks that were considered less important. Most had established daily routines for meals, studies/work and leisure time, and recognized the value of doing so to live a good life. As one participant said, “It becomes sort of an instruction in a way. Like a Lego-catalogue (. . .) I like that.” Having a set of everyday activities was also described as a way of taking care of own mental health:

Being occupied is the only thing that helps. If I am bored or don’t have anything to do, I either make stupid choices, or I get worse mentally. So I have to have something that makes the brain occupied. And that is creative things, and gaming.

In sum, the participants described a trial-and-error approach in finding different helpful strategies to compensate for being in challenging environments. These strategies were described as important for daily functioning, and thus contributing to better QoL.

### Theme 4: deriving meaning and purpose from interests and passions

Even if we did not ask specifically about interests, it became evident that interests had an essential influence on everyday living for the participants. Many participants described *interests as a way of spurring friendships (subtheme 4.1)*. Being focused on the same interests, made it easier to build connections and maintain friendships over time, and this had, for some, made a positive difference in the transition to senior high school, as mentioned in Theme 1. Friendships could also be made in the virtual world, via the interest of gaming: “He gave me a virtual hat, and after that a friendship was made.”

*Interests also acted as a guide to education and job career (subtheme 4.2)*. Interests had, for many, been considered when planning the entrance to work in adult life. Technology was a common interest, leading many of the students to choose to study engineering:

I study computer science (. . .) civil engineering (. . .) I have quite a large interest-field, really, I find many things exciting. But I like to use a lot of time on the computer. Many of my interests are probably quite strongly connected to that.

The participants also described the necessity of *finding meaningful activities (subtheme 4.3)* in their lives, which they could enjoy. Interests were described as important for the ability to focus and being motivated for academic studies and work: “You have to find a job so you can support yourself, that is important, but it is also important to find something that *means* something (. . .) that is why I go for [studying] history (. . .) and writing books.”

### Theme 5: cautious optimism about their futures

When asked about how they pictured their lives in 10 years from now, some participants had their plans arranged, and some were more reluctant to have specific thoughts about the future. A common feature across participants was the desire to be responsible adults. They all wanted to participate in society in different ways. Participants expressed concerns about being limited in education and careers and were keen to *expand opportunities in work and social life (subtheme 5.1).*

Quite a few already had their job careers set based on their education. Others were still looking for direction. Being able to be a student, also meant that they had more time to mature and be independent: “that is why I study for a Masters, it was a bit too early to live by myself and be a grown-up.” Some had experienced limitations in choice of career, because of the autism diagnosis. For example, the possibility of being an exchange student and/or choosing a military career is ruled out, as admission rules exclude autistic people in general.

Despite social life being described by many as tiring and something that reduced the “social battery,” many expressed a desire for a social life in the future. One participant, who currently had very limited contact with others, still wanted to “try having someone close.” This exemplifies the vulnerability also described in Theme 1, where experiences of being seen as different and/or being bullied, might lead to being disconnected from peers in emerging adulthood. But, still, the wish to try it out is still there, which expresses optimism for the future.

Among the participants, there was *uncertainty about wanting a family (subtheme 5.2).* Most of the participants who already had a partner were confident that they wanted their own family. Some had concrete plans for marriage and having children. Some who did not have partners, were optimistic about having a family in the future, and described thoughts of family life as desirable. A few mentioned the possibility of adoption as an alternative if love-life failed, as the desire to be a parent was strong. Others were clear that they did not want a partner or children. They appreciated the heavy commitment involved, and thought it was important to manage life by oneself first. There was a common perception among the participants that having children was an enormous commitment, as one participant eloquently expressed: “It is very unfair to have children if you don’t really intend to do all the work.”

Even if the participants had mixed perceptions of how they pictured their futures, they were individually very precise in describing it. Having a good, future QoL was described as having control of one’s life, taking care of oneself and possibly others.

## Discussion

In this study, we sought to understand what a sample of autistic young adults, clinically referred in childhood, believe is important for QoL throughout the different life phases of childhood, adolescence and emerging adulthood, and what they perceive to be the challenges and barriers to the possibility of having a good QoL. We found that the participants described different factors important for QoL, such as securing solid relationships with family and peers, being in control of their everyday lives, and ensuring opportunities to pursue their particular interests. The different factors described by the participants in this study align well with the definition of QoL by the World Health Organization (WHO, 1998) and the eight domains of QoL outlined by Schalock (2000, 2004), especially with regard to the domains of interpersonal relations and social inclusion. In addition, participants described meaningful activities as important, which might be connected to the QoL domain of personal development. What seems to be specific for this group of young adults with autism, is the description of several barriers in society and the support system, including poor communication with supporters, bullying, and a lack of person–environment fit. Schalock (2000) emphasizes how the concept of QoL goes beyond the individual person. Discrimination practices in society, such as deprivation of rights, regarded as one of the QoL domains by Schalock (2004), can negatively influence QoL. As an example of this, our participants described being denied military service and the opportunity of being an exchange student. Such practices are likely to lead to feelings of being excluded and less valued in society (Milton & Sims, 2016). In sum, our findings shed light on what QoL is for people on the autism spectrum and which factors might promote or hinder a good QoL in young adulthood.

**Figure 1.**
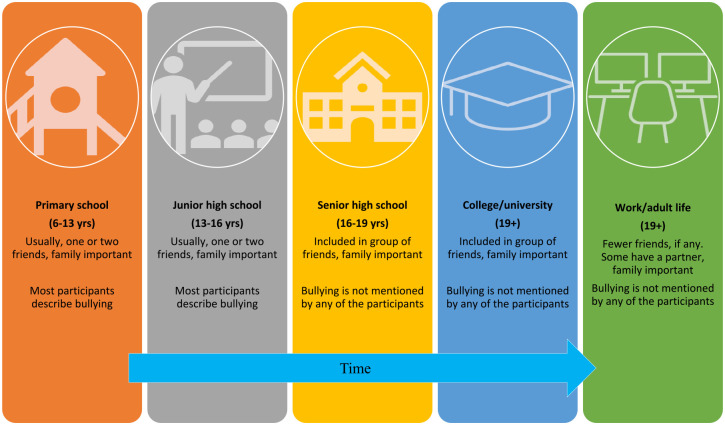
Development of relationship to family, peers, and being bullied, from childhood to emerging adulthood.

Our participants further described changes over time regarding their family connections, making friends, and perceived bullying. Support from family was felt to be particularly important, especially during childhood and through college/university. At younger ages, participants usually reported having one or two friends—and most described being the victims of bullying, too. Over time, during senior high school and college/university, the participants felt more included in groups of friends, and bullying was not mentioned. Those having social challenges during early school years, described becoming more socially secure and included when entering senior high school and college/university. These experiences are perhaps related to the educational context in Norway, where there is a distinct transition between junior high and senior high school.^1^ These findings align with another Norwegian qualitative study, showing that transition to senior high school can be a place where autistic youth can feel more included and do not experience bullying, despite having a previous history of bullying (Skafle et al., 2020). Our findings also echo those of another study, which reported that autistic students could fully “be themselves” in company of other peers during university, and they described friendships that developed to be more genuine at this time (Scott & Sedgewick, 2021). We believe that this development is important, as it brings hope for social inclusion in the period of emerging adulthood. We have illustrated this development in Figure 1.

Being connected to family and peers was described as important for QoL in this study, illustrated in Theme 1. This was also evident in a recent study by Pellicano, Brett, et al. (2022), where autistic people described the loss of social contact with others during the COVID-19 pandemic, and how this influenced their mental health negatively. It is vital to address the misconception that people on the autism spectrum do not wish to have a social life, also discussed by Jaswal and Akhtar (2019)—precisely as our participants suggested. Misconceptions about autism, combined with the consequences of bullying as a limitation for creating new friendships, seem to have influenced the childhood years negatively for some of our participants (see also Benevides et al., 2020; Tesfaye et al., 2023). While inclusive practices seem to be sparse in many societies, and appears difficult to change (Pellicano et al., 2018), our findings demonstrate the importance of promoting inclusive attitudes, particularly in schools, but also in society more broadly.

Misunderstandings extended beyond participants’ peer groups, however. As presented in Theme 2, our participants described experiences of mismatches in communication with teachers and practitioners in both schools and clinical settings. The mismatches were described as being misunderstood, misinterpreted, and infantilised. The participants also described having trouble understanding other’s intentions, as, for example, non-verbal language could be hard to interpret. These reports align with descriptions of the “double empathy problem,” where communication between autistic and non-autistic people is characterized by a lack of understanding from both parties (Milton, 2012). The ways in which non-autistic people could adapt their communication styles to better meet autistic communication styles is important to investigate in further studies.

Consistent with the findings presented here, participants in earlier qualitative studies describe animals as non-judgmental, sensitive, they enhance socialization, and they also act as stress-reducing helpers (Byström & Persson, 2015; Cheak-Zamora et al., 2018; Lam et al., 2020). The relation to pets could thus be seen as a means of meaning-making in everyday life, a way of getting around, having responsibility for someone, being more independent from parents, and promoting physical health. Physical health and independence are likely to be beneficial and lead to healthier aging for all people, and this could perhaps be even more crucial for potentially vulnerable groups, such as autistic people (Happé & Charlton, 2012). Enhancing opportunities to access, for example, dogs as therapeutic support, both in schools and in student housing, could perhaps contribute to better QoL for some autistic people.

Emerging adulthood is described as a period with less commitment, a self-focused age where there are opportunities to follow own interests, get to know oneself better and develop independence (Arnett, 2007). The participants described that, when in emerging adulthood, they had gradually used strategies that allowed them to become more in control of their lives. Our findings corroborate those of Cribb et al. (2019), who also found that autistic young adults reported wanting to be more in control and acknowledged that it takes time to mature into the adult role. However, in the phase of becoming independent, there is also the consideration of balancing energy, support from others and social connections to others, that might be more complex for autistic people than non-autistic people. The participants in our study specifically pointed out that mastering the balance of these factors was important for their QoL. For our participants, the objective dimensions of QoL regarding housing and education are supported by different financial programs in Norway. All students get support by the Norwegian state for living independently, and tuition is free at most major colleges and universities. We believe this leaves more room for the subjective QoL dimensions, such as personal development, and underscores the importance of contextual factors when describing QoL.

Spending time on interests and passions was for many an important factor for leading a meaningful life. Interests were described as a connection to friends, a gateway to academic and work career—and a source of enjoyment and meaning, also described in other studies (Grove et al., 2018; Wood, 2021). Spending time on special interests could also provide a sense of freedom and serve as a compass for social activities (Lizon et al., 2023), which might influence QoL positively. Yet, how interests influence QoL is not measured in any questionnaires about QoL that we are aware of. The findings from our research indicate that the impact of interests should perhaps be included as a topic in questionnaires designed to measure QoL for people with autism. The opportunities to immerse in interests seem vital for the QoL domain of personal development for autistic people, a topic discussed more thoroughly by Rapaport et al. (2023) and Lizon et al. (2023).

Generic QoL scales often set a standard for what an optimal outcome is, not always allowing for the nuanced views of autistic participants (Evers et al., 2022). This could result in a more negatively measured QoL than actually perceived. For example, as described in Themes 1 and 3, autistic people describe social connections as important but need them in moderation. Moderate levels of connections do not necessarily mean that QoL is perceived as poor, but it would be measured as poor on a generic QoL scale. As mentioned earlier, autism-specific QoL instruments have been developed to respond to the possible challenges of using generic QoL instruments (Ayres et al., 2018; McConachie et al., 2020). Evers et al. (2022) have discussed that generic and autism-specific QoL measures do not rule each other out, but should be used for different purposes. Autism-specific QoL measures could be crucial to inform services, evaluate interventions and especially enable active participation in health care (Backman et al., 2023). The study described in this article sheds light on subjective descriptions of QoL, using in-person interviews. We advocate for more research using qualitative measures to supplement quantitative measures. Perhaps when using qualitative measures, one can capture a more positive angle of self-worth and thus more positive descriptions of QoL for autistic people.

### Limitations

The participants in this study represent a clinical group diagnosed in childhood and early adolescence, within the average range of cognitive ability. Thus, they represent one subgroup of young adults with autism, and the results we have produced in this project may differ from results from other subgroups. Autistic girls were under-identified at the time when the LINEUP started in 2009. Consequently, women are heavily underrepresented in this study. While different general conceptualizations of QoL (Schalock, 2004; WHO, 1998) do not differentiate between male and female populations, more work is needed to be done to understand QoL in autistic people of different gender identities.

There might also be several other contextual factors in understanding the constructs of QoL described in this study. Emerging adulthood is characterized by self-focus and exploration. This might be reinforced by a welfare society like Norway, which has especially good student financial support systems. As described in Theme 5, some participants explained that being a student for a longer time gave more time to prepare for adult life. Autistic students have the right to use two extra years to complete senior high school in Norway, making it five years instead of three. Some of the participants had used this right. Also, since tuition is usually free, some of the participants in this study had completed two different bachelors and then decided on a masters. This could explain why so many were students. The Norwegian state’s financial support for students, could explain the high rate of participants living outside the family home. These limitations must be taken into consideration for analytic generalization and transferability of the results to other cultural contexts.

## Conclusion

The participants described wanting to have close connections to others but were aware of balancing this, as it drained their “social battery.” Future interventions should focus on how professionals can help autistic people establish or secure connections to people/animals, as this seems essential for having a good QoL. Also, opportunities to immerse oneself in one’s interests appeared to be important for perceived QoL. Teachers and clinicians should therefore be aware of the possibilities in harnessing interests, as it could lead to more meaningful social, academic, and work life. Future research and interventions should also look further into what the communication barrier between autistic people and professionals consists of, and how this could be improved. For many of the participants, adult life was something they still prepared for in different ways, emphasizing the term emerging adulthood. As one of the participants stated when picturing his future adult life: “One is perhaps never really ready.”

## Supplemental Material

sj-docx-1-aut-10.1177_13623613241245578 – Supplemental material for “I have to charge my social battery”: Perspectives from autistic young adults on Quality of Life Supplemental material, sj-docx-1-aut-10.1177_13623613241245578 for “I have to charge my social battery”: Perspectives from autistic young adults on Quality of Life by Elisabeth Øverland, Åshild Lappegard Hauge, Stian Orm, Merete Glenne Øie, Erik Winther Skogli, Elizabeth Pellicano and Per Normann Andersen in Autism

## Notes

1. In Norway, primary schools and junior high schools are run by municipalities. Senior high schools are run by counties, and college/universities are run by the state.
